# FDSS-Based DFT-s-OFDM for 6G Wireless Sensing

**DOI:** 10.3390/s23031495

**Published:** 2023-01-29

**Authors:** Lu Chen, Jianxiong Pan, Jing Zhang, Junfeng Cheng, Luyan Xu, Neng Ye

**Affiliations:** 1 School of Cyberspace Science and Technology, Beijing Institute of Technology, Beijing 100081, China; 2 School of Information and Electronics, Beijing Institute of Technology, Beijing 100081, China; 3 Science and Technology on Communication Networks Laboratory, Shijiazhuang 050081, China; 4 China Academy of Electronic and Information Technology, Beijing 100041, China; 5 Laboratory of Electromagnetic Space Cognition and Intelligent Control, Beijing 100083, China

**Keywords:** ISAC, DFT-s-OFDM, FDSS, estimation error, ambiguity function, PAPR

## Abstract

Integrated sensing and communications (ISAC) is emerging as a key technology of 6G. Owing to the low peak-to-average power ratio (PAPR) property, discrete Fourier transform spread orthogonal frequency-division multiplexing (DFT-s-OFDM) is helpful to improve the sensing range and suitable for high-frequency transmission. However, compared to orthogonal frequency-division multiplexing (OFDM), the sensing accuracy of DFT-s-OFDM is relatively poor. In this paper, frequency-domain spectral shaping (FDSS) is adopted to enhance the performances of DFT-s-OFDM including sensing accuracy and PAPR by adjusting the correlation of signals. Specifically, we first establish a signal model for the ISAC system, followed by the description of performance indicators. Then, we analyze the influence of amplitude fluctuation of frequency domain signals on sensing performance, which shows the design idea of FDSS-enhanced DFT-s-OFDM. Further, a FDSS-enhanced DFT-s-OFDM framework is introduced for ISAC, where two types of FDSS filters including a pre-equalization filter and an isotropic orthogonal transform algorithm (IOTA) filter are designed. The simulation results show that the proposed scheme can obtain about 4 dB performance gain in terms of sensing accuracy over DFT-s-OFDM. In addition, FDSS-enhanced DFT-s-OFDM can significantly reduce PAPR and improve the power amplifier efficiency.

## 1. Introduction

ISAC systems can perceive the physical characteristics of surroundings while transmitting communication information, which realizes the mutual enhancement of communication and sensing [1]. Approaching 6G, many new services continue to emerge, such as smart cities, smart homes, and autonomous driving. These intelligent services put forward more demands for the integration of sensing and communication [2,3,4]. Moreover, in 6G, wireless communication and sensing will have more similarities in the operating spectrum, hardware structure, and signal processing [5], which provides a further opportunity to integrate the communication and sensing. ISAC will be the emerging technology of 6G, which is characterized by multi-dimensional awareness and ubiquitous communication capability.

The ISAC waveform is an important part in the research of ISAC. It can realize the compatibility between sensing and communication, as well as an efficient utilization of resources. Owing to its multi-carrier characteristics and high spectrum utilization [6,7], OFDM has been regarded as a suitable ISAC waveform, which can directly add the sensing function to carry out the sensing detection process. There are considerable researches for ISAC waveform based on OFDM, for example, the robust ISAC waveform based on OFDM inspired by information theory [8]. An OFDM chirp waveform diversity for co-designed radar-communication system [9]. ISAC waveform based on the combination of phase coding and OFDM [10,11].

However, the main challenge of ISAC waveform based on OFDM lies in its high PAPR, which will cause nonlinear distortion and reduce the sensing range [12]. The concept of constant envelope orthogonal frequency-division multiplexing (CE-OFDM) is proposed to reduce the PAPR of the OFDM signal [13]. The idea is to implant the OFDM signal into the phase of constant envelope signal, but it will sacrifice a lot of spectral efficiency. Another better method to solve the problem of high PAPR of OFDM is the combination of DFT expansion with OFDM. By DFT expansion, OFDM has single-carrier characteristics which can reduce the PAPR. DFT-s-OFDM is more suitable for broadband transmission [14], as well as has higher power-amplification efficiency to reduce nonlinear distortion and improve the sensing range, which make it a better candidate for 6G sensing.

Unfortunately, the introduction of DFT will affect the sensing accuracy of OFDM. A lot of effort has been paid to optimizing the sensing performance. From the aspect of transceiver algorithm, refs. [15,16] proposed an approximated maximum likelihood radar parameter estimation algorithm to optimize the sensing performance for ISAC system based on OFDM. However, this will cause complexity on the transceiver, which is not suitable for small and intensive equipment. As for the waveform design, a multi-frequency complementary phase-encoded OFDM signal is proposed to promote the ambiguity function property by adjusting the phases of multiple symbols [17]. However, the communication information can only be modulated on the whole phase of OFDM. Subsequently, a direct-sequence spread-spectrum modulation method is proposed to optimize the ambiguity function of the overall signal [18,19]. Although this method can obtain good ambiguity function performance, the trade-off between communication and sensing performance is not clear.

All together, most schemes simply change the signal distribution by encoding or modulating the communication information, and rarely utilize the filter design. Time–frequency domain filters, such as FDSS [20], prototype filters, and shaping filters, can effectively improve the performance of the waveform by changing the time–frequency domain characteristics, and can be flexibly designed for specific sensing or communication properties. For example, the schemes of pruned DFT-spread filter bank multicarrier (FBMC) [21,22], FFT-based spectrally efficient frequency-division multiplexing (SEFDM) [23,24], and more compact faster-than-Nyquist (FTN) signals [25] have different tradeoffs between PAPR, BER, and spectrum efficiency (SE). Frequency-domain spectral shaping on DFT-s-OFDM for SE and PAPR performance has been proposed [26,27]. Exploiting FDSS, a framework for chirp-based communications, is proposed in [28]. However, it is rare that works focus on the optimization of sensing performance. Hence, utilizing the filter design to jointly optimize communication and sensing performance is worth consideration for ISAC systems.

Finally, it should be noted that alternative modulation or physical-layer approaches, such as FBMC, SEFDM, and FTN signals, have been proposed in the literature, with different tradeoffs between spectrum localization, resource allocation flexibility, multiantenna support, power-efficiency, and transceiver processing complexity. However, in most cases, these methods would cause high system complexity and call for a larger redesign of the 5G NR physical-layer specifications, and are thus not explicitly considered in our scheme.

In this paper, we propose an ISAC waveform which adopts FDSS to enhance the sensing performance of DFT-s-OFDM by adjusting the correlation of signals, as well as reduce the PAPR of DFT-s-OFDM. FDSS is a kind of modulation technology which uses a spectrum shaping sequence to effectively shape the waveform in a frequency domain. Specifically, we first establish a signal model for the ISAC system, and illustrate two sensing performance indicators, that is, ambiguity function and estimation error. Then, we investigate the ISAC waveform’s structure based on OFDM and DFT-s-OFDM, followed by the sensing performance comparison. Through the comparison, we can conclude that the smooth waveform helps to improve the sensing performance by weakening the effect of noise enhancement. On this basis, a framework of FDSS-enhanced DFT-s-OFDM for ISAC is introduced, and two methods, a pre-equalization filter and IOTA filter, are proposed as the FDSS filters to shape the DFT-s-OFDM waveform. Finally, through the simulation results, we observe that the proposed scheme can obtain about 4 dB performance more gain in terms of sensing accuracy than DFT-s-OFDM and outperform than other shaping methods. Meanwhile, the proposed scheme has the lower ambiguity function sidelobe, which means the enhanced resolution. Further, we study the effect of FDSS on PAPR performance. Compared with DFT-s-OFDM, FDSS can effectively reduce PAPR.

The main contributions of this paper are summarized as follows:

- We propose an FDSS-enhanced DFT-s-OFDM framework for ISAC to jointly improve the sensing and communication performance. Different from most works that only use FDSS to reduce the communication performance, we aim to enhance the sensing performance of DFT-s-OFDM by adjusting the correlation of signals, as well as reducing the PAPR of DFT-s-OFDM further.
- We propose the design idea of an FDSS filter which improves sensing accuracy by smoothing the signal amplitude and weakening the effect of noise enhancement. Inspired by this idea, we develop a FDSS filter based on pre-equalization of frequency-domain signals. It can significantly reduce the fluctuation of signals so as to weaken the noise enhancement caused by the channel estimation process. This way, the performance in terms of sensing accuracy can be improved. To our best knowledge, no one has proposed such scheme in the previous works.
- We evaluate the performance of the FDSS-enhanced DFT-s-OFDM waveform from both sensing and communication aspects, including range estimation accuracy, ambiguity function performance, and BER and PAPR performance, as well as perform the necessary comparison with other methods to verify the effectiveness of the proposed design.

The remainder of this paper is organized as follows. In Section 2, we establish the signal model for the ISAC system and describe the performance indicators. In Section 3, we introduce the OFDM and DFT-s-OFDM sensing waveform, followed by the sensing performance comparison. In Section 4, we propose the FDSS-enhanced DFT-s-OFDM waveform for ISAC, where two FDSS filters and three corresponding detection algorithms are considered. Simulation results are presented in Section 5. Section 6 concludes this paper.

## 2. System Model

In this section, the signal model for ISAC system is briefly described. Then, the performance indicators for the sensing signal are presented.

### 2.1. Signal Model for Integrated Sensing and Communication

**Figure 1:**
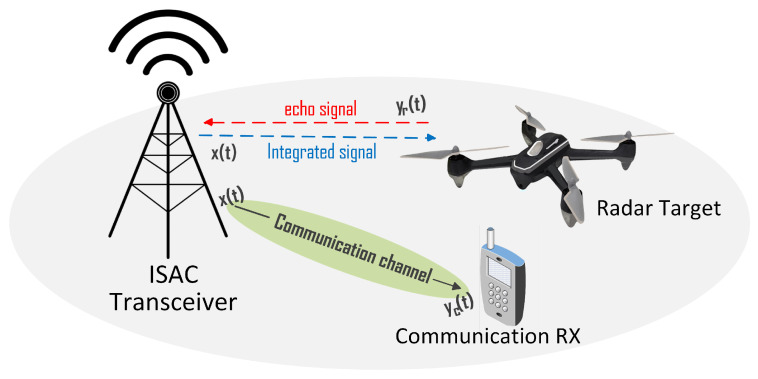
ISAC system model.

As shown in Figure 1, we consider that the system contains one ISAC transmitter (base station) and *P* ISAC targets (vehicle), and use the same set of waveform and equipment to realize the integration of sensing and communication. Specifically, the communication and sensing processes share the same transmitter. The receiving processing of communication part is completed at the vehicle, and the receiving processing of the sensing part is completed at the base station.

The ISAC signal x(t) is transmitted from the ISAC transceiver, and *P* echo signals scatter from targets with different distances $R_{p}$, and the corresponding relative velocities $v_{p}$, ∀p=1,2,…,P are received by the ISAC transceiver. Accordingly, the delay of the target is $\tau_{p}=2R_{p}/c$ and the Doppler shift is $f_{dp}=2f_{c}v_{p}/c$, where *c* is the speed of light and $f_{c}$ is the carrier frequency of the transmitted signal. Then, the received echo signal $y_{p}\left(t\right)$ of the object with ditances $R_{p}$ can be expressed as [15]

(1) $$y_{p}\left(t\right)=x\left(t-\tau_{p}\right)e^{j2\pi f_{dp}t}+n\left(t\right)$$

where n(t) is additive Gaussian white noise. Through the processing of echo signals, parameters such as time delay and frequency offset are extracted to realize accurate estimation of target attributes such as distance and speed.

### 2.2. Performance Indicators

Some indicators are required to measure the sensing performance of the ISAC system. The performance indicators are introduced as follows, mainly including ambiguity function and estimation error.

#### 2.2.1. Ambiguity Function

As an important tool for sensing waveform design and analysis, the ambiguity function can characterize the waveform and corresponding matched filter. When two targets are monitored by the system at the same time, if the positions and velocities of the two objects differ greatly, they can be easily distinguished. However, if the positions and distances of these two objects are very close, it will bring challenges to the system. The ambiguity function is used to measure the ability of the system to distinguish between objectives. By analyzing the ambiguity function of the sensing waveform, we can obtain the resolution, measurement accuracy, and ambiguity of the sensing system under optimal matched filter processing.

There are many ways to define ambiguity functions. The following definitions are adopted in this paper [29].

(2) $$\chi \left(\tau ,f_{d}\right)=\int_{-\infty }^{+\infty }y_{p}\left(t\right)y_{p}^{*}\left(t-\tau \right)e^{i2\pi f_{d}t}dt$$

where $y_{p}\left(t\right)$ is the echo signal, $y_{p}^{*}\left(t\right)$ represents the conjugate of $y_{p}\left(t\right)$. τ and $f_{d}$ are the time delay difference and doppler frequency shift difference between two objects’ echo signal, respectively. As $|\chi \left(\tau ,f_{d}\right)|$ decreases faster with τ and $f_{d}$ increasing, the resolution of sensing system is stronger, the ambiguity is lower, and the two objects are easier to distinguish.

#### 2.2.2. Estimation Error

Root mean square error (RMSE) is employed as the performance measure for the sensing system, which can be obtained by calculating the standard deviation between the actual distance and the predicted distance. The smaller the value of RMSE, the higher the accuracy of distance estimation. The formula of RMSE is provided as follows.

(3) $$\mathrm{RMSE}=\sqrt{\frac{1}{N}\sum_{i=1}^{n}{\left(Y_{i}-f\left(x_{i}\right)\right)}^{2}}$$

where $Y_{i}$ is the actual value, such as the distance $R_{p}$ and the relative velocity $v_{p}$ of the target object, $f(x_{i})$ is the corresponding estimated value, *N* is the number of data and ∑ is the total number of values.

In addition to the above sensing performance, ISAC system also needs to ensure the communication performance, such as spectrum-efficiency and bit-error-rate, as well as considering the power amplifier efficiency, PAPR and other indicators to reduce nonlinear distortion and improve the range of sensing and communication.

## 3. OFDM-Based Waveforms for Sensing

In this section, we mainly introduce the ISAC waveforms structure based on OFDM and DFT-s-OFDM, followed by the sensing performance comparison.

### 3.1. Vanilla OFDM-Based ISAC Waveform

The basic principle of OFDM is to divide the high-speed serial data into multiple parts, and each part of the data is carried by a subcarrier. In this way, the symbol rate of the data will be much lower, as well as enhancing the anti-fading and anti-multipath ability of the system. The OFDM signal is transmitted in the form of multiple symbol blocks, which is suitable for the sensing mode.

The traditional integrated OFDM signal pulse consists of one OFDM symbol, which does not consider the transmission of communication information, leading to low communication data rates and difficult synchronization. Therefore, current researchers [15,16] mostly consider a pulse formed by several consecutive OFDM symbols with communication information, and all OFDM symbols in a pulse form a frame or a complex frame, as to realize the communication function in a pulse.

On this basis, we consider the continuous ISAC waveform based on OFDM symbol as (4). Specifically, each sensing pulse consists of $N_{s}$ OFDM symbols, and each OFDM symbol includes $N_{c}$ subcarriers. The time duration of one OFDM symbol is $T_{s}$, and subcarrier spacing is Δf=1/T. Then the OFDM-based ISAC signal x(t) can be described as follows [16].

(4) $$x\left(t\right)=\sum_{m=0}^{N_{s}-1}\sum_{n=0}^{N_{c}-1}a_{m,n}e^{j2\pi n\Delta f(t-mT_{s})}\mathrm{rect}\left(\frac{t-mT_{s}}{T_{s}}\right)$$

where $a_{m,n}$ represents the communication information on the *n*-th subcarrier of the *m*-th OFDM symbol. The function rect(.) describes a rectangular function, which is equal to 1 for $0<t⩽T_{s}$, and 0 otherwise. Then, the received echo signal $y_{p}\left(t\right)$ can be expressed as

(5) $$\begin{array}{cc} y_{p}\left(t\right) & =x\left(t-\tau_{p}\right)e^{j2\pi f_{dp}t}+n\left(t\right)\\ \; & =\sum_{m=0}^{N_{s}-1}\sum_{n=0}^{N_{c}-1}a_{m,n}e^{j2\pi n\Delta f(t-\tau_{p}-mT_{s})}e^{j2\pi mf_{dp}t}\mathrm{rect}\left(\frac{t-mT_{s}-\tau_{p}}{T_{s}}\right)+n\left(t\right) \end{array}$$

**Figure 2:**
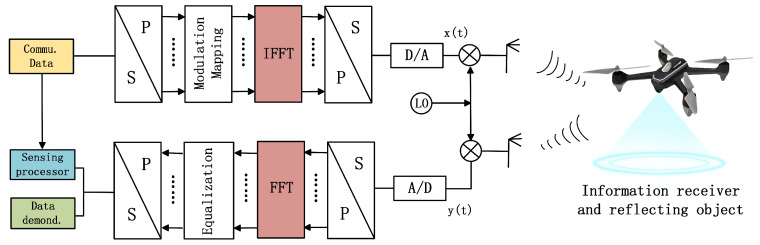
ISAC system structure based on OFDM signal.

The ISAC system based on OFDM signal is shown in Figure 2. At the transmitter, the communication data is divided into parallel streams, and mapped onto the modulation symbol sequence. By inverse fast Fourier transform (IFFT), and a subsequent parallel-to-serial conversion, the OFDM-based ISAC signal x(t) is obtained. After the digital to analog conversion, the signal is transmitted. In the receiver, the received modulation symbols are recovered from the received signal y(t) by a fast Fourier transform (FFT) operation, while the estimated range and speed information of the sensing target are obtained after sensing processor. The classical detection algorithms mainly includes maximum likelihood estimation algorithm, DFT/IDFT algorithm and so on.

### 3.2. DFT-s-OFDM-Based ISAC Waveform

DFT-s-OFDM and OFDM have similar implementation structures. The main difference is that DFT-s-OFDM has an DFT calculation process before subcarrier mapping, resulting in the difference in signal transmission form. Specifically, DFT-s-OFDM first performs the *M*-point DFT on the data block. Then the output data is used as the continuous input of OFDM modulator, which is realized by *N*-point IDFT. Normally *N* > *M*, the unused inputs of the IDFT are set to zero. Finally, similar to OFDM, it is better to insert CP into each block. On this basis, we consider the ISAC waveform based on DFT-s-OFDM symbol as (6).

(6) $$x\left(t\right)=\sum_{m=0}^{N_{s}-1}\sum_{n=0}^{N-1}b_{m,n}e^{j2\pi n\Delta f(t-mT_{s})}\mathrm{rect}\left(\frac{t-mT_{s}}{T_{s}}\right)$$

where

(7) $$\begin{array}{c} b_{m,n}=\left\{\begin{array}{c} \sum_{k=0}^{M-1}d_{k}e^{-j2\pi nk/M},0\leq n<M\\ \;\;\;\;0\;\;\;,M\leq n<N \end{array}\right. \end{array}$$

where $d_{k}$ represents the communication information [30]. Then, the received echo signal $y_{p}\left(t\right)$ can be rewritten as

(8) $$\begin{array}{cc} y_{p}\left(t\right) & =x\left(t-\tau_{p}\right)e^{j2\pi f_{dp}t}+n\left(t\right)\\ \; & =\sum_{m=0}^{N_{s}-1}\sum_{n=0}^{N-1}b_{m,n}e^{j2\pi n\Delta f(t-\tau_{p}-mT_{s})}e^{j2\pi mf_{dp}t}\mathrm{rect}\left(\frac{t-mT_{s}-\tau_{p}}{T_{s}}\right)+n\left(t\right) \end{array}$$

OFDM modulates the information of the symbol itself to the orthogonal subcarrier, while DFT-s-OFDM modulates the spectrum information of M continuous sampling values in the original sequence to the orthogonal subcarrier. OFDM simultaneously transmits the values of multiple symbols in parallel in a symbol period, and each symbol occupies an independent subchannel, which is equivalent to multiple subchannels transmitting data at the same time. However, DFT-s-OFDM uses all subchannels to transmit the information of the same symbol, which is equivalent to only one single carrier transmitting data, i.e., DFT-s-OFDM signal has the single-carrier nature.

Compared with OFDM, the main advantage of DFT-s-OFDM is the low PAPR, which means the possibility of improving the efficiency of the power amplifier. Simultaneously, DFT-s-OFDM has frequency division multiple access (FDMA) with flexible bandwidth allocation, low complexity, and high-quality equalization in the frequency domain.

### 3.3. Performance Comparisons on OFDM-Based Range Estimation

The sensing performance of the ISAC waveform is mainly considered from sensing range and sensing accuracy. For the sensing range, compared with OFDM, the low PAPR of DFT-s-OFDM can raise the power amplifier efficiency, which is beneficial to improving the sensing range. For the sensing accuracy, we simulate the RMSE for OFDM and DFT-s-OFDM distance estimation under QPSK modulation, using the maximum likelihood estimation algorithm.

**Figure 3:**
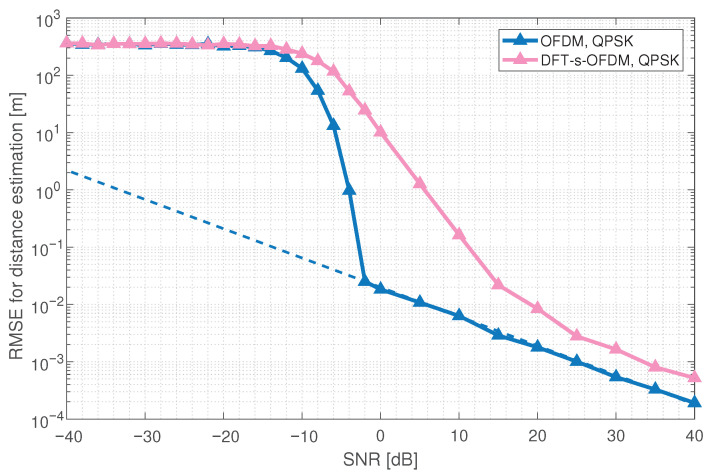
Comparison of the RMSE for distance estimation between OFDM and DFT-s-OFDM.

**Figure 4:**
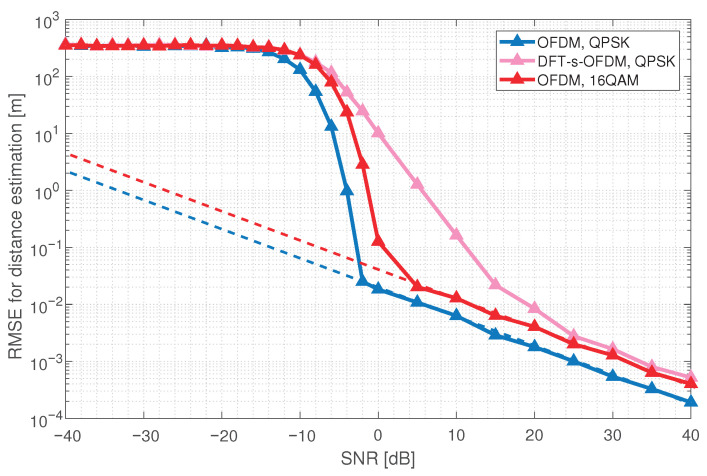
Comparison of the RMSE for distance estimation between 16 QAM and QPSK.

As shown in Figure 3, the RMSE for OFDM distance estimation first remains unchanged with the increase of SNR, then falls sharply, and finally decreases linearly, coinciding with CRB. Compared with OFDM, the distance estimation performance of DFT-s-OFDM is 5–10 dB worse. Simulation results show that the sensing accuracy of DFT-s-OFDM is worse than OFDM, and the improvement of DFT-s-OFDM for sensing is necessary. Moreover, under the same configurations, we simulate the RMSE for OFDM distance estimation with different modulation modes. As shown in Figure 4, the distance estimation performance of OFDM under 16 QAM modulation is 5 dB worse than that under QPSK modulation.

**Figure 5:**
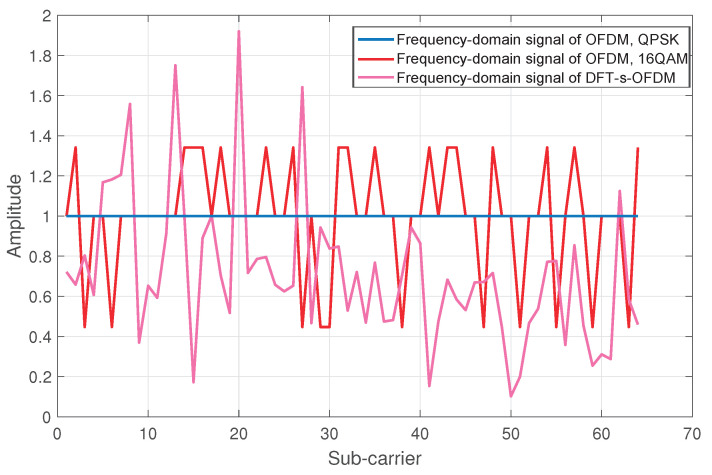
Comparison of OFDM signal under different modulations.

Further, the comparison between OFDM signal waveforms under different modulation modes and DFT-s-OFDM signal is shown in Figure 5. Compared with QPSK, the OFDM signal waveform under 16 QAM modulation is more fluctuant, and the DFT-s-OFDM signal has the most serious amplitude fluctuation, i.e., the worst sensing performance.

It is worth noting that, compared with the OFDM signal under QPSK modulation, the amplitude of the OFDM signal under 16 QAM modulation and DFT-s-OFDM signal are not unimodular. Therefore, the noise enhancement for the subcarriers will be caused after the equalization processing at the receiver, where the amplitude of signals are less than 1. Large ripples of the signal waveform degrade the sensing performance due to the noise enhancement with equalization processing.

From the above analysis emerges the idea that the smoother the frequency domain of the signal waveform, the better the sensing performance. Therefore, the DFT-s-OFDM signal can be shaped in frequency domain to improve its sensing performance.

## 4. Proposed FDSS-Enhanced DFT-s-OFDM Design for Sensing

In this section, we propose a framework for FDSS-enhanced DFT-s-OFDM, which aims to enhance the sensing performance of DFT-s-OFDM by adjusting the relevance of signals, as well as reduce the PAPR of DFT-s-OFDM. Firstly, the FDSS technology is introduced briefly. On the basis, the DFT-s-OFDM signal based FDSS is described, followed by two FDSS filters and three corresponding detection algorithms.

### 4.1. Briefs of FDSS

A set of discrete time domain signals are converted into analog continuous signals after passing through the digital to analog conversion module (DAC). The PAPR of the analog continuous signal has a certain relationship with the correlation between the set of discrete time domain signals [20]. To explain this theory concretely, we propose a set of discrete time domain signals y(n) and a set of discrete data d(n). Then, yd(n) is obtained as

(9) y(n)⊗d(n)=yd(n)

If d(n) is a set of designed weight coefficient series, the correlation between the adjacent data of yd(n) will be better than that between the adjacent data of y(n). Therefore, the PAPR of yd(n) output signals after DAC will be lower than that of y(n). On this basis, by adjusting the weight coefficient d(n), the correlation between symbols can be adjusted to improve the PAPR performance.

According to the convolution theorem, the convolution operation of two time-domain signals can be equivalent to the multiplication operation of them in the frequency domain. Therefore, by transforming a set of discrete time domain data into discrete frequency domain data after DFT, and then multiplying the data with the designed spectrum shaping sequence, the reshaped sequence can be obtained. Owing to the low complexity of point multiplication operation, this sequence reshaping technology operates better in the frequency domain, which is called frequency-domain spectral shaping (FDSS).

In this way, introducing FDSS into signal can adjust the correlation between signals to effectively reduce the PAPR, so as to enhance its communication performance. Simultaneously, FDSS can adjust the waveform amplitude of signals to weaken the impact of noise enhancement, so as to improve the sensing performance.

### 4.2. FDSS-Enhanced DFT-s-OFDM ISAC Waveform

**Figure 6:**
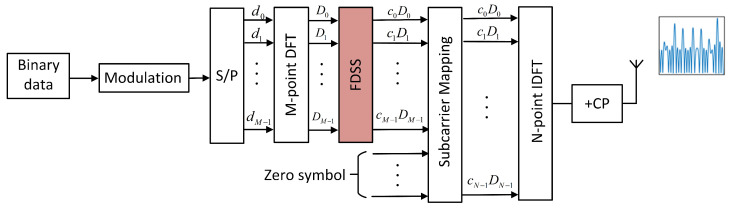
A DFT-s-OFDM transmitter model with a special FDSS filter.

The FDSS-enhanced DFT-s-OFDM symbol can be implemented according to the framework shown in Figure 6. First, operate the *M*-point DFT on the modulation symbol sequence, i.e., $\left[d_{0},d_{1},\ldots ,d_{M-1}\right]$. Then, multiply each term of DFT output sequence by corresponding Fourier coefficient, i.e., FDSS. Finally, operate the *N*-point inverse DFT (IDFT) on the shaped sequence padded with zero symbols. Accordingly, the FDSS-enhanced DFT-s-OFDM signal can be described as

(10) $$p\left(t\right)=\underset{N-\mathrm{point}\;\mathrm{IDFT}}{\underset{︸}{\sum_{n=0}^{N-1}\underset{\mathrm{Frequency}-\mathrm{domain}\;\mathrm{spectral}\;\mathrm{shaping}}{\underset{︸}{c_{n}\underset{M-\mathrm{point}\mathrm{DFT}}{\underset{︸}{\sum_{k=0}^{M-1}d_{k}e^{-j2\pi n\frac{k}{M}}}}}}e^{j2\pi n\Delta ft}}}$$

where $c_{n}$ is the weight factor of DFT signal, which can be derived from the Fourier series [28]. By adjusting the form of the Fourier series base, DFT-s-OFDM signals can generate a pseudo-Chip pulse of different shapes, such as linear, triangular, and sinusoidal, which can meet the diverse needs of different sensing scenes. The FDSS-enhanced DFT-s-OFDM ISAC signal x(t) can be rewritten as

(11) $$x\left(t\right)=\sum_{m=0}^{N_{s}-1}\sum_{n=0}^{N-1}d_{m,n}e^{j2\pi n\Delta f(t-mT_{s})}\mathrm{rect}\left(\frac{t-mT_{s}}{T_{s}}\right)$$

where,

(12) $$\begin{array}{c} d_{m,n}=\left\{\begin{array}{c} c_{n}\sum_{k=0}^{M-1}d_{k}e^{-j2\pi nk/M},0\leq n<M\\ \;\;\;\;0\;\;\;\;\;,M\leq n<N \end{array}\right. \end{array}$$

where $d_{k}$ represents the communication information. Then, the received echo signal $y_{p}\left(t\right)$ can be rewritten as

(13) $$\begin{array}{cc} y_{p}\left(t\right) & =x\left(t-\tau_{p}\right)e^{j2\pi f_{dp}t}+n\left(t\right)\\ \; & =\sum_{m=0}^{N_{s}-1}\sum_{n=0}^{N-1}d_{m,n}e^{j2\pi n\Delta f(t-\tau_{p}-mT_{s})}e^{j2\pi mf_{dp}t}\mathrm{rect}\left(\frac{t-mT_{s}-\tau_{p}}{T_{s}}\right)+n\left(t\right) \end{array}$$

**Figure 7:**
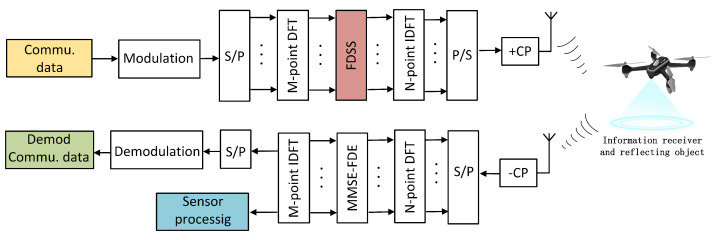
ISAC system structure based on FDSS-enhanced DFT-s-OFDM.

On the basis of Figure 2, an ISAC system based on FDSS-enhanced DFT-s-OFDM is shown in Figure 7. At the transmitter, the DFT signals are superimposed into a form similar to chirp pulses by weighting the DFT samples, which can realize the sensing and detection functions on the basis of the original communication model. The receiver uses a single-tap minimum mean square error (MMSE) frequency-domain equalization (FDE) to remove the impact of the channel. FDSS is applied between DFT and IFFT processes, which is essentially the shaping of frequency domain signals after DFT. Therefore, for the actual receiver, the FDSS coefficient can be regarded as a part of the channel frequency response and estimated through the channel estimation process [28]. In this way, the impact caused by the introduction of FDSS can be eliminated after MMSE-FDE. From this aspect, a typical DFT-s-OFDM receiver can demodulate the FDSS-enhanced DFT-s-OFDM symbol without any changes. In addition, if the FDSS coefficients are available a priori at the receiver, the receiver can perform better because they do not need to be estimated.

In the following section, we focus on the two important parts of FDSS-enhanced DFT-s-OFDM ISAC system, i.e., the FDSS filter design and the detection algorithm for range estimation.

### 4.3. FDSS Filter Design

Inspired by the comparison between the OFDM sensing performance under different modulation modes, we introduce two implementation methods of FDSS, that is, the pre-equalization filter and the IOTA filter. Both the two FDSS filter design methods make the frequency domain signal amplitude tend to be uniform so as to improve the system sensing performance.

#### 4.3.1. Pre-Equalization Filter

Frequency domain equalization starts from the frequency domain of the system and directly processes the amplitude frequency characteristics and phase frequency characteristics of the system in the frequency domain, that is, adding frequency domain filters to make them reach the desired transfer function.

**Figure 8:**
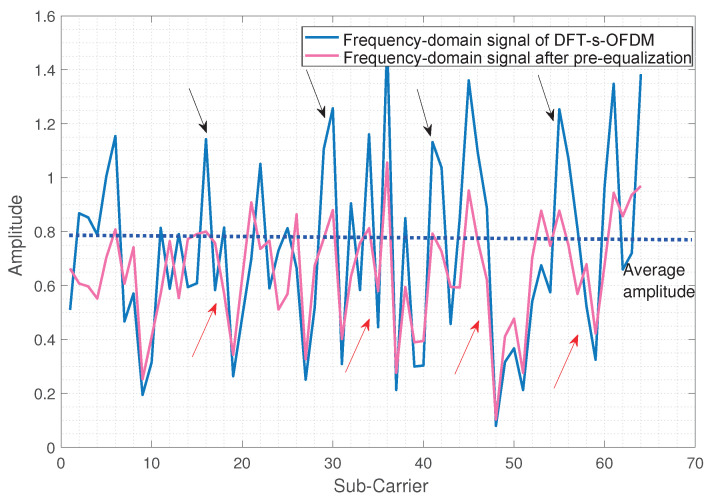
Subcarrier amplitude of DFT-s-OFDM after equalization.

In this section, we adopt the pre-equalization filter as a method to realize FDSS. Specifically, we design a filter to equalize the amplitude of the DFT-s-OFDM signal, which aims to change the amplitude of the frequency domain signal close to the average value as much as possible. For each DFT-s-OFDM symbol, we define the frequency domain sequence as $m=\{m_{1},m_{2},\ldots ,m_{N}\}$, where *N* is the number of subcarriers, and calculate the average amplitude value E(m). Then, taking the average amplitude value as a benchmark, the part beyond the average value is compressed, and the part below the average value is increased to minimize the variance of the symbol amplitude. In this way, we obtain the pre-equalization sequence $m_{0}$ as

(14) $$\begin{array}{c} m_{0}=f\left(m\right)=\left\{f\left(m_{1}\right),f\left(m_{2}\right),\ldots ,f\left(m_{N}\right)\right\},\; \end{array}\;\left\{\begin{array}{c} f\left(m_{i}\right)=\xi_{a}m_{i},\;m_{i}>E\left(m\right)\\ f\left(m_{i}\right)=\xi_{b}m_{i},\;m_{i}<E\left(m\right) \end{array}\right.$$

where $\xi_{a}$ is the compression ratio, and $\xi_{b}$ is the increase ratio. The frequency domain amplitudes of DFT-s-OFDM signal and the equalized signal are shown in Figure 8. Compared with the DFT-s-OFDM signal, the frequency domain amplitude of equalized signal become more uniform.

#### 4.3.2. IOTA Filter

IOTA is a filter function obtained by the time-frequency quadratic orthogonal operation of Gaussian function [31]. Therefore, the IOTA filter has obtained the orthogonality while maintaining the good time-frequency localization (TFL) of Gaussian function. The approximate time-domain IOTA function can be expressed as [32]:

(15) $$\xi_{\tau_{0}}\left(t\right)=\frac{1}{2}\sum_{k=0}^{K-1}\left\{{\bar{d}}_{k,v_{0}}\left[h_{EGF}\left(t+\frac{k}{v_{0}}\right)+h_{EGF}\left(t-\frac{k}{v_{0}}\right)\right]\right\}\times \sum_{l=0}^{K}\left[{\bar{d}}_{l,\tau_{0}}\cos\left(2\pi l\frac{t}{\tau_{0}}\right)\right]$$

where the parameter $\tau_{0}$ and $v_{0}$ are chosen to be $1/\sqrt{2}$ normally, and $h_{\mathrm{EGF}}\left(t\right)=2^{\frac{1}{4}}e^{-\pi t^{2}}$ is the EGF in the time domain. ${\bar{d}}_{k,v_{0}}$ is the IOTA coefficients, which can be expressed as:

(16) $${\bar{d}}_{k,v_{0}}=\sum_{q=0}^{Q-1}b_{k,q}\times e^{-\pi (2q+k)},0\leq k\leq K-1,0\leq q\leq Q-1$$

where *K* and *Q* are two parameters of the IOTA filter, generally, K=15 and Q=8.

**Figure 9:**
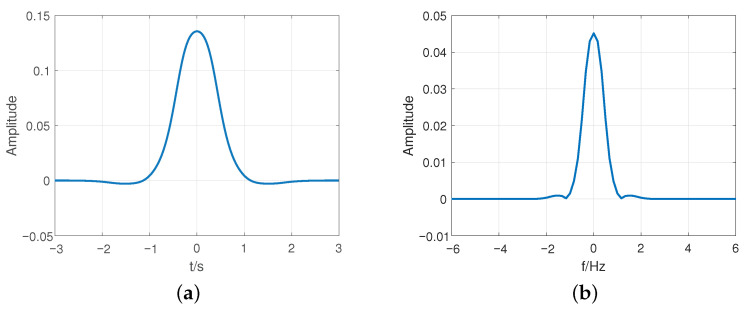
IOTA filter in time and frequency domain. (**a**) Time domain. (**b**) Frequency domain.

Figure 9 shows the IOTA filter function in the time and frequency domain. It can be observed that the IOTA filter has a relatively ideal TFL and good isotropic properties in the time-frequency domain. Since it has ideal TFL characteristics, IOTA is able to reduce the out-of-band (OOB) emission [33,34], PAPR, and inter subcarrier interference (ISI). Therefore, we select the IOTA filter with TFL properties as an FDSS sequence to adjust the correlation between DFT-S-OFDM symbols, so as to improve the sensing performance and reduce PAPR, while still meeting the OOB emission and pass band signal quality requirements.

### 4.4. Detection Algorithm of FDSS-Enhanced DFT-s-OFDM for Range Estimation

The detection algorithm also affects the sensing performance of the ISAC waveform. Here we consider three corresponding detection algorithms, including DFT detection algorithm, time domain correlation algorithm, and maximum likelihood estimation.

#### 4.4.1. DFT Method

Due to the signal characteristics of DFT-s-OFDM, it is easy to have a range estimation by DFT detection. First, the received signal is divided into $N_{s}$ blocks according to the symbol period. Then, we remove the CP and obtain the *m*-th received block. At the output of the OFDM demultiplexer, we can obtain the received complex modulation symbols, which contains the range and velocity information of the target object. After element-wise divisions, the range information can be expressed as follows [16].

(17) $$k_{r}\left(n\right)=e^{-j2\pi n\Delta f\left(2R/c_{0}\right)},0\leq n\leq N-1$$

where *N* is the number of subcarriers.

Therefore, the range information is readily obtained by taking an IDFT of $k_{r}\left(n\right)$, which can be expressed as

(18) $$r\left(k\right)=\frac{1}{N}\sum_{n=0}^{N-1}e^{-j2\pi n\Delta f\left(2R/c_{0}\right)}e^{j2\pi nk/N}$$

#### 4.4.2. Time Domain Correlation Estimator

Time domain correlation refers to the correlation processing between the local code, such as PN code, M code, and other codes with good auto-correlation, and the received signal. Through the correlation value we can judge the strength of the correlation, and then determine the correlation position, i.e., the acquisition position and synchronization position. The essence of the time-domain correlation calculation is to calculate the correlation value by summing the product of the local code and received signal. The time-domain correlation algorithm is expressed as follows.

(19) $$\rho \left(\tau \right)=\int_{-\infty }^{+\infty }x\left(t\right)y\left(t-\tau \right)dt$$

where x(t) is the transmitted signal, and y(t−τ) is the received echo signal.

#### 4.4.3. Maximum Likelihood Estimator

Maximum likelihood estimation is a widely used parameter estimation method. The idea of maximum likelihood estimation is that for a given observation data *x*, we hope to find the parameter $\theta^{*}$ which can generate observation data with maximum probability from all parameters $\theta_{1},\theta_{2},\cdots ,\theta_{n}$ as the estimation result. The estimated parameter $\theta^{*}$ shall satisfy

(20) $$L\left(\theta^{*}|x\right)=p\left(x|\theta^{*}\right)\geq p\left(x|\theta \right)=L\left(\theta |x\right),\theta =\theta_{1}\ldots ,\theta_{n}$$

In the actual operation, we regard the parameter θ to be estimated as a variable, and calculate the probability function p(x∣θ). Then we find the parameter θ which can maximize the probability function to generate the observation data *x*. The parameter θ is expressed as follows, which can be solved by finding the derivative equal to 0 [15].

(21) $$\theta^{*}=\arg\max_{\theta }p\left(x|\theta \right)$$

## 5. Simulation Results

To verify the effectiveness of the proposed design, we simulate the performance of FDSS-enhanced DFT-s-OFDM waveform from many aspects, including distance estimation accuracy, ambiguity function performance, and BER and PAPR performance. The simulation framework is according to Figure 7. At the transmitter, the binary communication data are divided into parallel streams, and mapped onto complex-valued QPSK symbols. Then, DFT is performed on the modulation symbol sequence. By weighting the DFT samples with the FDSS coefficient, the DFT sequences are superimposed into a form similar to chirp pulses. After IFFT and a subsequent parallel-to-serial conversion, the FSDD-enhanced DFT-s-OFDM ISAC signal is obtained. Then, time delay and Gaussian noise are added to simulate the transmission process under an ideal channel. In the receiver, the received modulation symbols are recovered from the received signal by a fast Fourier transform (FFT) operation. DFT/IDFT algorithm is adopted as the detection algorithms to obtain the range estimation. The simulation is implemented by MATLAB software with a Dell EMC XE2420 Server. Basic parameter configuration is shown in Table 1.

### 5.1. Range Estimation Accuracy for FDSS-Enhanced DFT-s-OFDM

**Figure 10:**
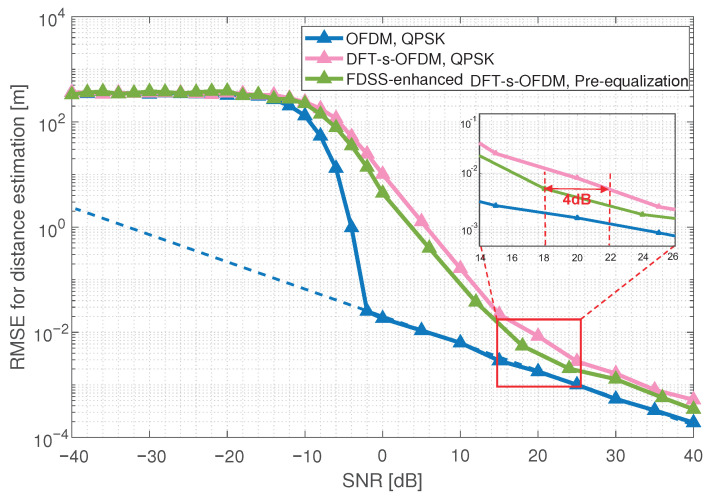
Sensing performance of FDSS-enhanced DFT-s-OFDM with pre-equalization.

We simulate the RMSE for the FDSS-enhanced DFT-s-OFDM distance estimation under a pre-equalization filter and IOTA filter. As shown in Figure 10, the RMSE for the equalized DFT-s-OFDM distance estimation is significantly better than baseline. A gain of about 2 dB can be obtained, and even under certain SNR range (14 dB to 26 dB), the gain can reach 4 dB. The simulation result shows that by shaping the DFT-s-OFDM waveform to the average amplitude, the pre-equalization filter can make the amplitude of the equalized DFT-s-OFDM waveform as uniform as possible, and then can improve the sensing performance of DFT-s-OFDM signal.

**Figure 11:**
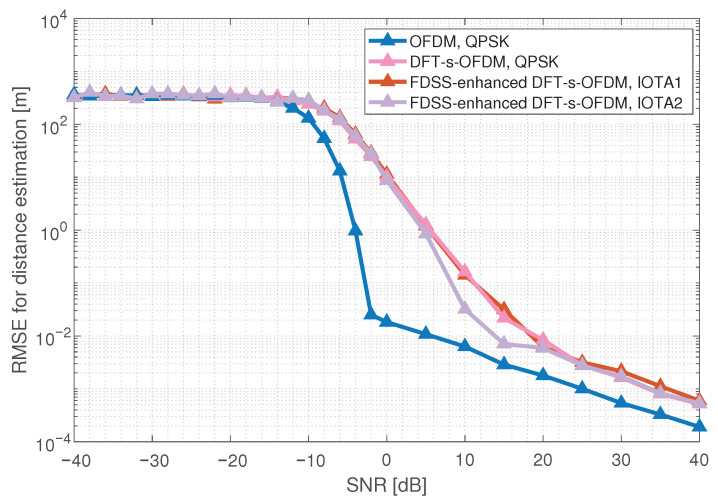
Sensing performance of FDSS-enhanced DFT-s-OFDM with IOTA.

The RMSE for the DFT-s-OFDM distance estimation under different IOTA are shown in Figure 11, where the waveform of IOTA2 is smoother than that of IOTA1 and performs better. From the results, we can observe that FDSS-enhanced DFT-s-OFDM based on the IOTA filter has the same range sensing performance as DFT-s-OFDM, and can obtain a degree of gain under certain SNR.

We can conclude that the two implementation methods of FDSS both can improve the sensing performance of DFT-s-OFDM waveform. As discussed in Section 3.3, equalizing the amplitude of the signal in the frequency domain can help to improve the sensing accuracy of the distance, which is reflected in both the pre-equalization and flattening IOTA2.

### 5.2. Ambiguity Function Performance

**Figure 12:**
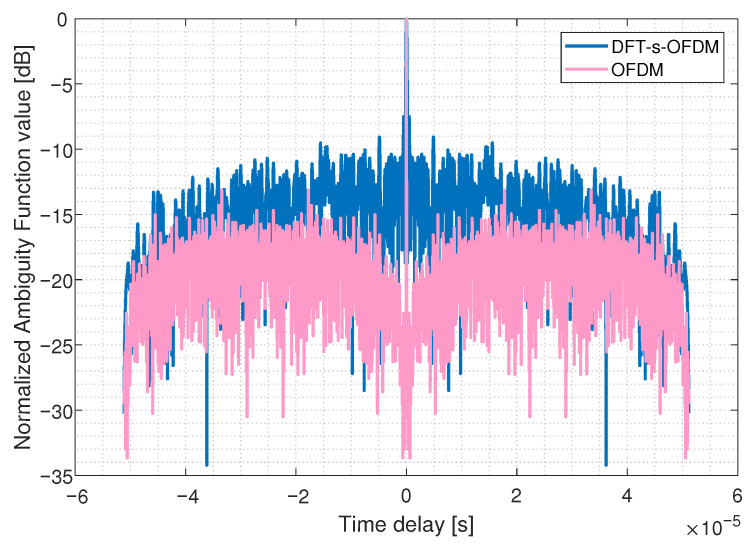
Comparison of ambiguity functions between DFT-s-OFDM and OFDM.

**Figure 13:**
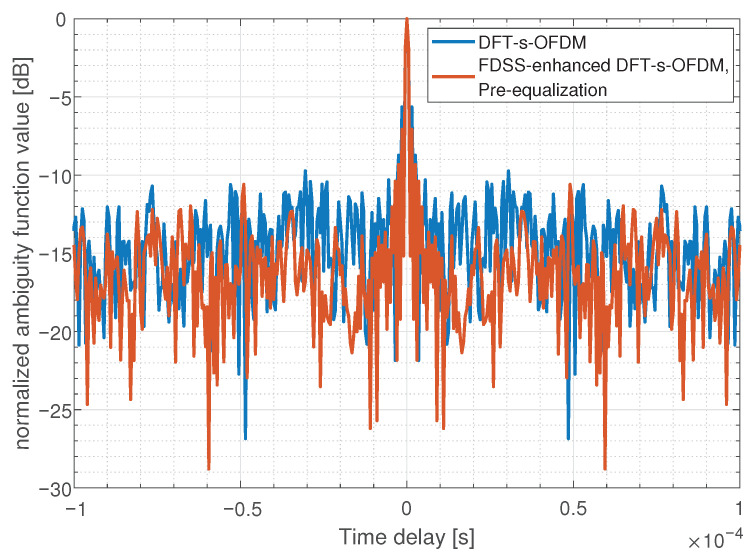
Comparison of ambiguity functions between DFT-s-OFDM and FDSS-enhanced DFT-s-OFDM with pre-equalization.

We simulate the ambiguity function to verify the change of FDSS-enhanced-DFT-s-OFDM sensing performance. The steeper the main peak of the ambiguity function, the stronger the resolution of the sensing target. As Figure 12 and Figure 13 shows, the time-delay domain ambiguity function sidelobe of OFDM is lower than that of DFT-s-OFDM, which means OFDM has better sensing performance. It is consistent with our previous results. After pre-equalization, the ambiguity function of FDSS-enhanced DFT-s-OFDM waveform has a lower sidelobe, which helps to improve the sensing accuracy.

**Figure 14:**
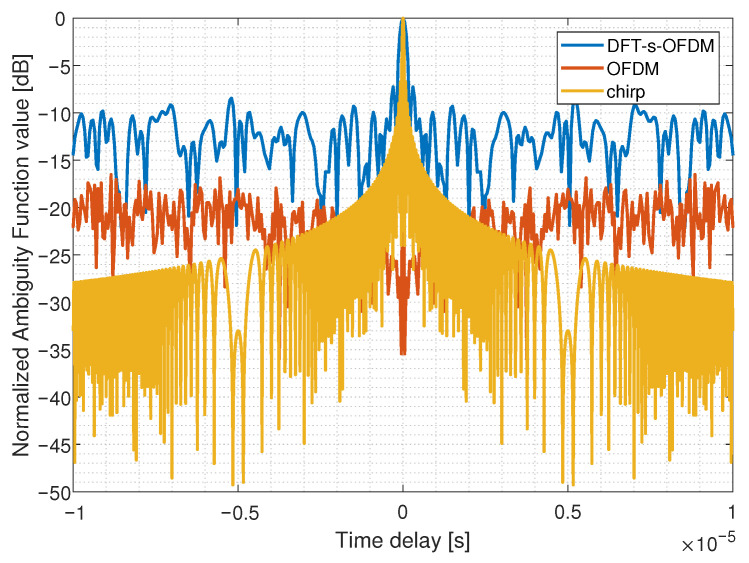
Comparison of ambiguity functions among DFT-s-OFDM, OFDM and chirp.

**Figure 15:**
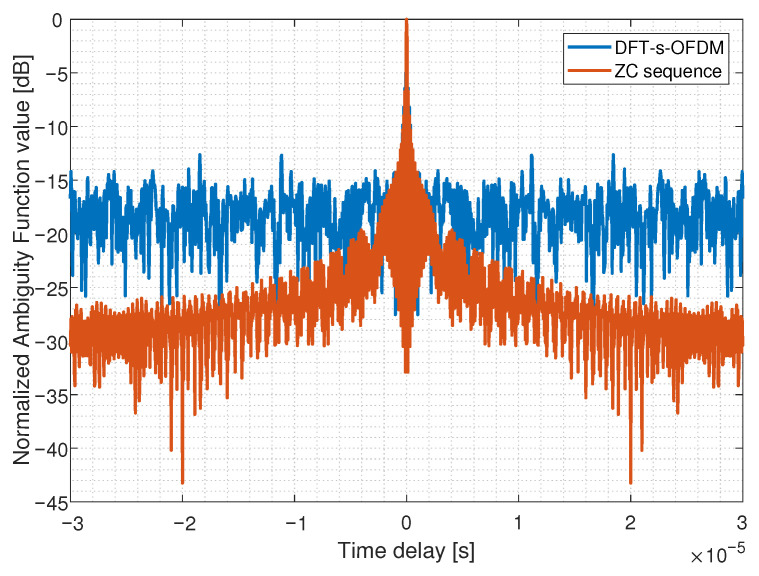
Comparison of ambiguity functions between DFT-s-OFDM and ZC.

Moreover, we compare the ambiguity functions between DFT-s-OFDM and other sequences such as chirp and Zadoff–Chu (ZC) sequences. As an ideal sensing waveform, chip has a lower ambiguity function sidelobe than both OFDM and DFT-s-OFDM, which is shown in Figure 14. For ZC sequences, which have constant amplitude, good autocorrelation and cross-correlation, the ambiguity function sidelobe is lower than DFT-s-OFDM, as shown in Figure 15. Chirp and ZC sequences have constant envelope and average amplitude, so they have better sensing performance, which is similar to our design idea.

### 5.3. BER and PAPR Comparisons

**Figure 16:**
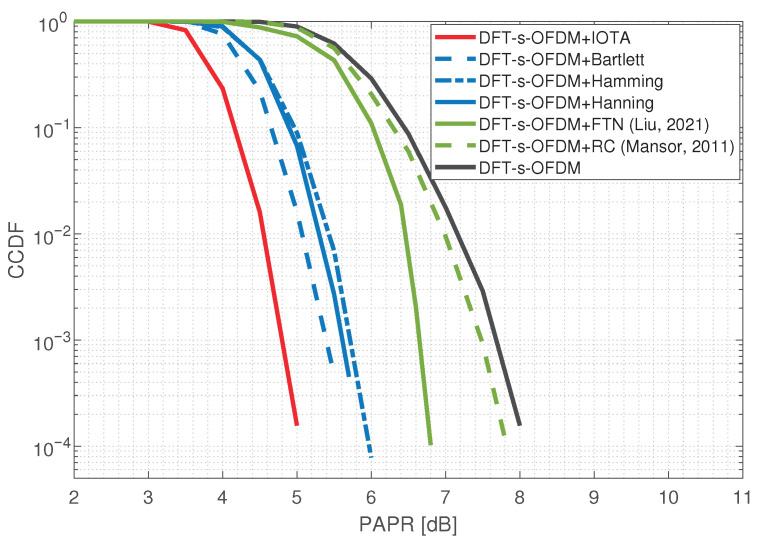
Effect of FDSS on PAPR performance, including the results of FTN [26] and RC [27].

In addition, we also simulate the influence of FDSS on PAPR and BER performance. Under the condition of QPSK, we compare the proposed scheme with other FDSS filters such as Bartlett filter, Hann filter and Hamming filter, as well as methods used in other articles such as FTN [26] and raised cosine spectrum shaping [27]. The simulation results of PAPR performance are shown in Figure 16. It can be observed that FTN and RC shaping methods can obtain PAPR gains of 1 dB and 0.2 dB, respectively. The methods based on other filters can obtain PAPR gains of about 2 dB. The FDSS scheme based on IOTA can obtain PAPR gains of 3 dB. Compared with other schemes, FDSS-enhanced DFT-s-OFDM based on an IOTA filter has a better PAPR performance without a loss of sensing performance.

**Figure 17:**
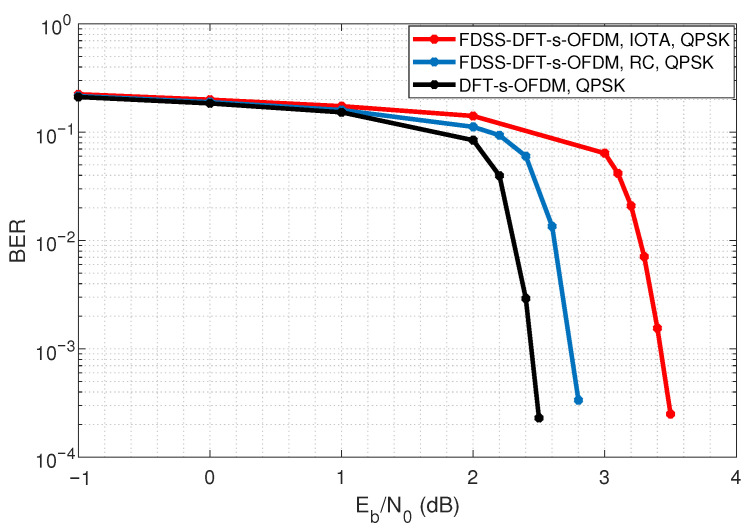
Effect of FDSS on BER performance.

As shown in Figure 17, the introduction of FDSS in DFT-s-OFDM causes a certain degree of loss of BER performance. Under the condition of QPSK, the SNR loss of FDSS scheme based on RC is less than 0.5 dB, and the SNR loss of IOTA is less than 1 dB.

## 6. Conclusions and Future Work

In this paper, we proposed an FDSS-enhanced DFT-s-OFDM waveform for an ISAC system to improve both sensing and communication performance. Firstly, we established a signal model for the ISAC system, followed by illustrating the corresponding performance indicators. Through the simulation of RMSE for OFDM distance estimation under different modulation methods, we observed that the amplitude fluctuation of the ISAC waveform in frequency domain can affect its sensing performance, owing to the noise enhancement. Specifically, the smoother waveform in the frequency domain causes less noise enhancement, which makes the sensing performance better. On this basis, we proposed a framework of FDSS-enhanced DFT-s-OFDM, which utilized FDSS to shape the DFT-s-OFDM waveform in frequency domain to make the distribution of amplitude more uniform. Simultaneously, we designed the FDSS filters based on a pre-equalization filter and IOTA filter, both of which were shown to significantly smooth the frequency domain signals. Finally, the simulation results show that FDSS-enhanced DFT-s-OFDM can obtain a performance gain of about 4dB on sensing accuracy. Meanwhile, the ambiguity function sidelobe of FDSS-enhanced DFT-s-OFDM waveform is lower than that of DFT-s-OFDM, which means the resolution of sensing is enhanced. Further, we studied the effect of FDSS on signal PAPR performance. Compared with DFT-s-OFDM, FDSS can effectively reduce PAPR.

Moreover, several issues need to be considered in the future. For example, the BER performance of our scheme is not good, and FDSS coefficient setting is not flexible. As a powerful means to realize the complex optimization, deep neural networks (DNN) can be used for more intelligent FDSS coefficient setting, so as to improve the sensing and communication performance jointly for ISAC system.

The main benefit of the proposed design is that it provides a method to effectively improve the performance of ISAC waveform, including sensing accuracy and PAPR property, by changing the time–frequency domain characteristics of the waveform with a filter. In addition, the proposed design can be carried out to meet different sensing or communication performance requirements with high design flexibility, which provides a potential design idea for complex and diverse wireless sensing in 6G application scenarios [35,36,37].

## Figures and Tables

**Table 1:** Basic parameter configuration.

Parameter	Numerical Value
Data modulation	QPSK
Carrier frequency	5.89 GHz
Bandwidth	10 MHz
Number of subcarriers	64
Symbol period	6.4 μs
Carrier interval	156.25 kHz
Symbol number	1000
CP number	16
Longest distance	240 m
Antenna gain	100
RCS	1 m^2^
Transceiver distance	20 m
Channel model	AWGN
Channel coding	LDPC (code rate 658/1024)

## Data Availability

Not applicable.
